# *Hantaviridae*: Current Classification and Future Perspectives

**DOI:** 10.3390/v11090788

**Published:** 2019-08-27

**Authors:** Lies Laenen, Valentijn Vergote, Charles H. Calisher, Boris Klempa, Jonas Klingström, Jens H. Kuhn, Piet Maes

**Affiliations:** 1 KU Leuven, Department of Microbiology and Immunology, Rega Institute for Medical Research, Zoonotic Infectious Diseases Unit, 3000 Leuven, Belgium; 2 Department of Laboratory Medicine, University Hospitals Leuven, 3000 Leuven, Belgium; 3 Colorado State University, Fort Collins, CO 80523, USA; 4 Biomedical Research Center, Slovak Academy of Sciences, 845 05 Bratislava, Slovakia; 5 Center for Infectious Medicine, Department of Medicine Huddinge, Karolinska Institutet, Karolinska University Hospital, SE-141 86 Stockholm, Sweden; 6 Integrated Research Facility at Fort Detrick, National Institute of Allergy and Infectious Diseases, National Institutes of Health, B-8200 Research Plaza, Frederick, MD 21702, USA

**Keywords:** classification, *Bunyavirales*, DEmARC, *Hantaviridae*, hantavirid, hantavirus, nomenclature, orthohantavirus, taxonomy

## Abstract

In recent years, negative-sense RNA virus classification and taxon nomenclature have undergone considerable transformation. In 2016, the new order *Bunyavirales* was established, elevating the previous genus *Hantavirus* to family rank, thereby creating *Hantaviridae*. Here we summarize affirmed taxonomic modifications of this family from 2016 to 2019. Changes involve the admission of >30 new hantavirid species and the establishment of subfamilies and novel genera based on DivErsity pArtitioning by hieRarchical Clustering (DEmARC) analysis of genomic sequencing data. We outline an objective framework that can be used in future classification schemes when more hantavirids sequences will be available. Finally, we summarize current taxonomic proposals and problems in hantavirid taxonomy that will have to be addressed shortly.

## 1. Introduction

Recent environmental, animal, and plant metagenomic studies have resulted in an avalanche of viral genomic sequencing data, vastly expanding the known virus biodiversity [1,2,3,4,5,6,7,8]. These advancements in the field of virus discovery led to a striking discrepancy between the number of potential new viral taxa described in literature and the number of officially recognized taxa by the International Committee on Taxonomy of Viruses (ICTV) [9]. Reasons for the backlog in official classification were not only the sheer number of novel viruses but also the absence of described biological properties of these viruses beyond genomic sequencing data and sequence-inferred characteristics. In the past, most ICTV Study Groups were reluctant to create new taxa in the absence of additional information on phenotypic virus properties, such as host range, antigenic relatedness, and virion morphology [9,10]. A consensus statement endorsed by the ICTV Executive Committee, explicitly permitting classification based on genomic sequence data alone (while still encouraging the acquisition of additional data) has opened the door to a much-needed reformation of the taxonomy of many virus families [9] and, therefore, an improved official depiction of the evolutionary relationships in the virosphere [11].

Hantaan virus and its immediate relatives have monopartite, trisegmented, negative-sense RNA genomes and produce enveloped virions [12]. After their first isolation in the 1970s [13,14], these viruses have been assigned to a distinct genus, *Hantavirus*, included in the family *Bunyaviridae* [15,16]—historically the largest negative-sense RNA virus family encompassing viruses infecting vertebrates, invertebrates, and plants [17]. Recent studies resulted in the discovery of numerous viruses similar, but distantly related, to those already classified in this family [4,7,8,18,19,20,21,22,23,24,25,26,27,28,29,30]. These discoveries led to the taxonomic promotion of the family as an order, *Bunyavirales*, in 2017 [31], and the continuous expansion of that order [32]. In 2017, the former bunyavirid genus *Hantavirus* was promoted within *Bunyavirales* to become the current *Hantaviridae* family [31,32].

Until 2007, all newly discovered hantavirids, with the exception of the shrew-borne Thottapalayam virus [13], were detected in or isolated from rodents (Mammalia: Rodentia). Since then, the recognized host range of hantaviruses has expanded to a large number of insectivores (Mammalia: Eulipotyphla), in particular shrews and moles, and to bats (Mammalia: Chiroptera), and even reptiles and fish [7,33,34,35,36,37]. This host-range expansion has been accompanied by an even larger expansion in hantavirid genetic diversity. Phylogenetic ancestral host reconstruction implicated that hantavirids have evolved over a considerable time span, leading to divergent hantavirid clades [38,39,40,41]. Large-scale PCR screening and detection of hantavirid genomes in a large number of hosts have unfortunately also resulted in large number of incomplete genome sequences [42]. This obvious increase in genetic hantavirid diversity accompanied by incomplete datasets has confounded the establishment of an all-encompassing hantavirid classification.

The ninth ICTV report of 2011 states the following demarcation criteria for hantavirid species classification:

“Species are usually found in unique ecological niches, i.e., in different primary rodent/insectivore reservoir species. Species exhibit at least 7% difference in aa identity on comparison of the complete glycoprotein precursor and nucleocapsid protein sequences (there are some exceptions presumably caused by historically recent host-switching events). Species show at least four-fold difference in two-way cross neutralization tests. Species do not naturally form reassortants with other species”.

[17]

These criteria indirectly imply that hantavirid classification into a species requires knowledge of its natural host, significant coverage of the viral genome sequence, and virus isolation in culture. In addition, cross-neutralization experiments, typically requiring biosafety level 3 containment, should be performed. Given stringent criteria, not all hantavirid species listed in the ninth ICTV report actually meet the these criteria [17]. For a minority of hantavirids, isolates were not available. For three hantavirids, the M segment sequence was incomplete or unavailable. Furthermore, certain hantavirids can cross host species barriers in opposition to the first criterion that suggests that a distinct hantavirus should be associated with a unique ecological niche [43,44]. Moreover, not all hantavirids listed in the ninth ICTV report meet the second criterion that denotes a minimum amino acid difference of 7% in nucleocapsid (encoded by the small (S) genomic segment) and glycoprotein (encoded by the medium (M) genomic segment) amino acid sequences. Consequently, the second criterion was proposed to be changed to a difference of >10% amino acid differences of the nucleoprotein and >12% amino acid difference of the glycoprotein [45].

Taxonomy is a continuous process that needs to keep pace with virus discovery and novel methodologies. The taxonomy of *Hantaviridae* clearly requires a comprehensive overhaul. The rationale and methodology for the beginning of this overhaul, formulated in official ICTV taxonomic proposals (TaxoProps) 2016.023a-cM, 2017.006M, 2017.012M, and 2018.010M (https://talk.ictvonline.org/), is outlined in the next sections of this manuscript.

## 2. Materials and Methods

### 2.1. DEmARC Analysis for Hantaviridae

The analysis was limited to hantavirids for which coding-complete S and M segment sequences were available. The deduced amino acid sequences of the proteins encoded by these segments (nucleoprotein and glycoprotein, respectively) of all available tentative hantavirid sequences were downloaded from NCBI’s GenBank. A concatenated multiple sequence alignment was generated with MAFFT v7 [46]. Bayesian phylogenetic inference was conducted in BEAST 1.8.4 [47] using 20 independent runs that continued until adequate effective sample sizes (ESS > 200) were obtained. Independent runs were combined using LogCombiner 1.8.4 (BEAST) [47], employing a burn-in of 10%. A consensus tree was built using TreeAnnotator 1.8.4 (BEAST) [47] with the maximum clade credibility method and visualized in FigTree v1.4 [48]. This consensus tree was used as a guide tree for the DivErsity pArtitioning by hieRarchical Clustering (DEmARC) analysis [49,50]. Pairwise evolutionary distance (PED) values were calculated using a maximum-likelihood approach with a WAG substitution model in Tree-Puzzle. A PED cut-off value of 0.1 was used for species demarcation within *Hantaviridae*.

### 2.2. Phylogenetic Inference for the Bunyavirales

The polymerase amino acid sequences of significant representative members of *Bunyavirales* were extracted from NCBI’s GenBank. In addition, new sequences stemming from viruses likely to be related to order members, including Jiāngxià mosquito virus 2 (JMV-2) [4], were considered in the analysis. Multiple sequence alignment was performed with MAFFT v7 after which a Bayesian phylogenetic reconstruction was conducted with BEAST 1.8.4. Two independent Markov Chain Monte Carlo analyses were run until adequate ESS were obtained. A consensus tree was built employing a burn-in of 10% in TreeAnnotator 1.8.4.

## 3. Results

### 3.1. Change of Demarcation Criteria

To establish an impartial hantavirid classification that is easily reproducible and adheres to the consensus about the exclusive use of sequencing data, we abandoned the ninth ICTV report’s species demarcation criteria and instead applied a classification approach based solely upon genetic data. DEmARC analysis was used to objectively define classification ranks based upon PED [49] and to establish taxonomic revisions of *Hantaviridae* in consecutive years since 2017.

Ideally, sequence-based classification relies on complete or at least coding-complete genome sequences [51], which, in the case of hantavirids, would be sequences of the three genomic segments S, M, and large (L). Unfortunately, for a large number of hantaviruses, availability of coding-complete sequences is limited, and, in particular, L segment sequences are frequently missing. Because the coding sequence of a single genomic segment does not contain sufficient information to achieve meaningful classification, we used a multiple sequence alignment of concatenated amino acid sequences of the S and M segments. DEmARC analysis gave a frequency distribution of PED values of which a threshold of 0.1 gave an optimal clustering cost of zero. Consequently, this threshold was selected as a hantavirid demarcation criterium at the species rank. Genera are demarked by a PED-value threshold of 0.95. Subfamilies are demarked based on their distinct clustering in the maximum clade credibility tree (see Figure 3) and a PED-value threshold of 3.5.

**Figure 1:**
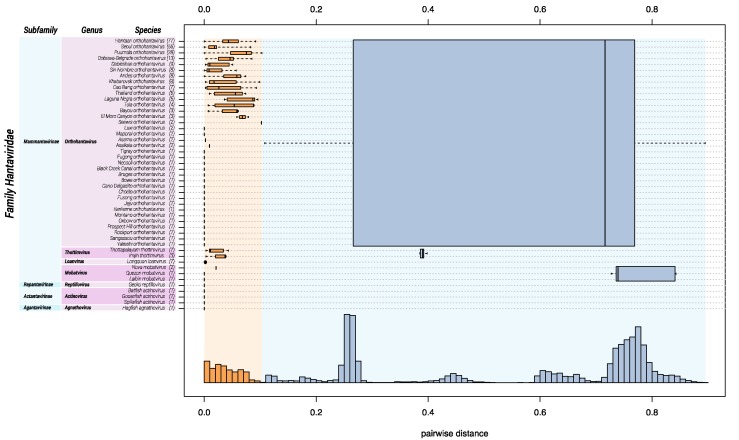
DEmARC analysis of the family *Hantaviridae*. Bayesian phylogenetic inference of hantavirid species is shown on the left. Hantavirids are classified into subfamilies, genera, and species based on DEmARC analysis (right). A frequency distribution (*y*-axis) of the PED values (*x*-axis) was plotted by species (orange), genus (blue), and subfamily (purple) demarcation. Boxplots and whiskers plot rank-specific PED distributions.

Based upon available sequence information in 2018, *Hantaviridae* can be divided into 4 subfamilies, 7 genera, and 47 species (Figure 1). “Classic” (bat-, mole-, shrew-, and rodent-borne) hantavirids were assigned to the subfamily *Mammantavirinae* in four genera: *Loanvirus*, *Mobatvirus*, *Orthohantavirus* and *Thottimvirus*. Fish- and reptile-borne hantavirids were assigned to three additional, monogeneric, subfamilies (*Actantavirinae*, *Agantavirinae* and *Repantavirinae*) [52].

### 3.2. Addition of New Taxa to Hantaviridae

Numerous new hantavirid species were incorporated into the ICTV-official taxonomy based on DEmARC analysis in 2017. Of these, 8 hantavirids have rodents as their natural hosts, whereas 3 newly discovered hantaviruses infect bats, 5 infect moles, and 8 infect shrews. Today, these viruses are distributed among the four mammantavirin genera *Loanvirus*, *Mobatvirus*, *Orthohantavirus* and *Thottimvirus* (Table 1) [52].

In 2018, viral metagenomics led to the discovery of new hantavirids in reptiles, jawless fishes (Agnatha), and ray-finned fishes (Actinopterygii) [7]. In line with the DEmARC analysis results, five additional hantavirid species were created in three genera: *Actinovirus* (*Actantavirinae*), *Agnathovirus* (*Agantavirinae*) and *Reptillovirus* (*Repantavirinae*). In addition, the complete genome sequences of 2 additional orthohantaviruses became available (Table 2) [32].

**Figure 2:**
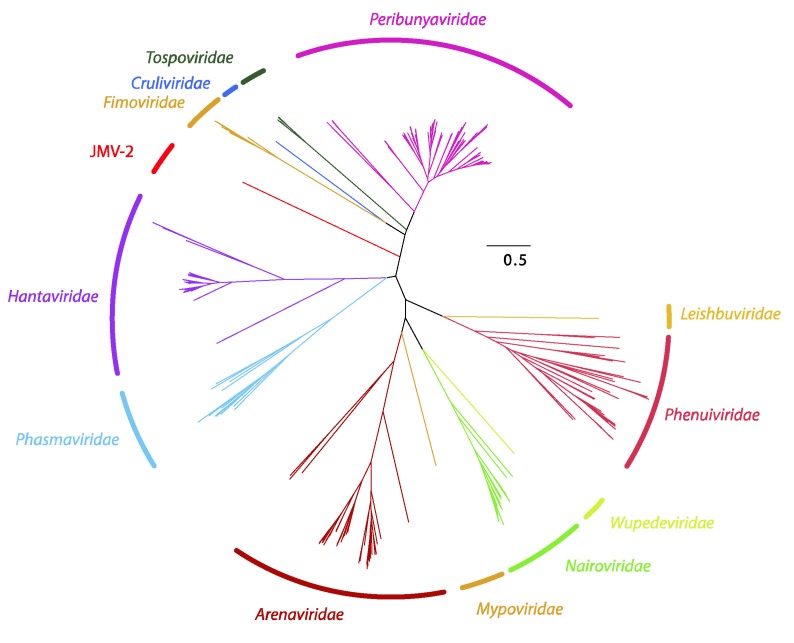
Bayesian inference of the L segment of *Bunyavirales*. A maximum clade credibility tree of the complete amino acid sequence of the protein encoded by the L segment of viruses belonging to *Bunyavirales*.

Using metagenomics, Jiāngxià mosquito virus 2 (JMV-2) was discovered. This virus is a highly divergent virus most closely (albeit very distantly) related to hantavirids [4] that has subsequently been described as the first mosquito-borne hantavirid [40]. However, phylogenetic analysis of the amino acid sequence of the coding-complete sequence of the JMV-2 L segment demonstrates that JMV-2 is divergent from all hantavirids and likely represents a novel family in *Bunyavirales* (Figure 2).

### 3.3. Abolishment of Hantavirid Taxa and Declassification of Hantavirids

From the introduction of new objective classification criteria based on sequence data, 8 previously recognized hantavirid species were abolished because they did not fulfill all criteria used for DEmARC analysis-based classification:

- *Amga orthohantavirus*: This species was abolished based upon insufficient differentiation from another species in DEmARC analysis. *Amga orthohantavirus* was established in 2017 for Amga virus (MGAV), which was discovered and sequenced in 2013 [38]. Since then, the coding-complete sequence of the S, M, and L genomic segment of Seewis virus (SWSV), detected in a Eurasian common shrew in 2007 [34], became available. DEmARC analysis demonstrated that Amga virus and Seewis virus belong to the same orthohantavirus species. Based upon the earlier discovery of Seewis virus, the species for both viruses became *Seewis orthohantavirus* in 2019 (Table 2) [32];
- *Isla vista hantavirus*: This species was abolished in 2017 based upon incomplete sequence data for the species member, Isla Vista virus (ISLAV) [73]. Only 1 complete S segment sequence, a partial M sequence, and no L segment sequences are available at this time. Our analyses using the incomplete data suggest that ISLAV represents a novel orthohantavirus species;
- *Muleshoe hantavirus*: This species was abolished in 2017 based upon incomplete sequence data for the species members, Muleshoe virus (MULV) [74]. Only 1 complete S segment sequence is available at this time;
- *New York hantavirus*: This species was abolished in 2017 based upon insufficient differentiation from another species in DEmARC analysis. The species member, New York virus (NYV), is highly similar to Sin Nombre virus (SNV, *Sin Nombre orthohantavirus*) in nucleoprotein and glycoprotein amino acid sequence comparisons, indicating that NYV is a SNV variant even though NYV and SNV can be distinguished by seroneutralization [75];
- *Rio Mamore hantavirus*: This species was omitted in 2017 based upon insufficient differentiation from another species in DEmARC analysis. The species member, Rio Mamoré virus (RIOMV) [76] is highly similar to Laguna Negra virus (LANV; *Laguna Negra orthohantavirus*) in DEmARC analysis and is now considered a LANV variant;
- *Rio Segundo hantavirus*: This species was abolished in 2017 based upon incomplete sequence data for the species member, Río Segundo virus (RIOSV) [77]. Only 1 complete S segment sequence is available;
- *Saaremaa hantavirus*: This species was abolished in 2017 based upon insufficient differentiation from another species in DEmARC analysis. The species member, Saaremaa virus (SAAV) [78], should be considered a member of the species *Dobrava-Belgrade orthohantavirus*; and
- *Topografov hantavirus*: This species was abolished in 2017 based upon insufficient differentiation from another species in DEmARC analysis. The species member, Topografov virus (TOPV) [79], is highly similar to Khabarovsk virus (KHAV) and, based upon the DEmARC analysis, should be considered as a KHAV variant.

### 3.4. Creation of Subfamilies and Genera within Hantaviridae

**Figure 3:**
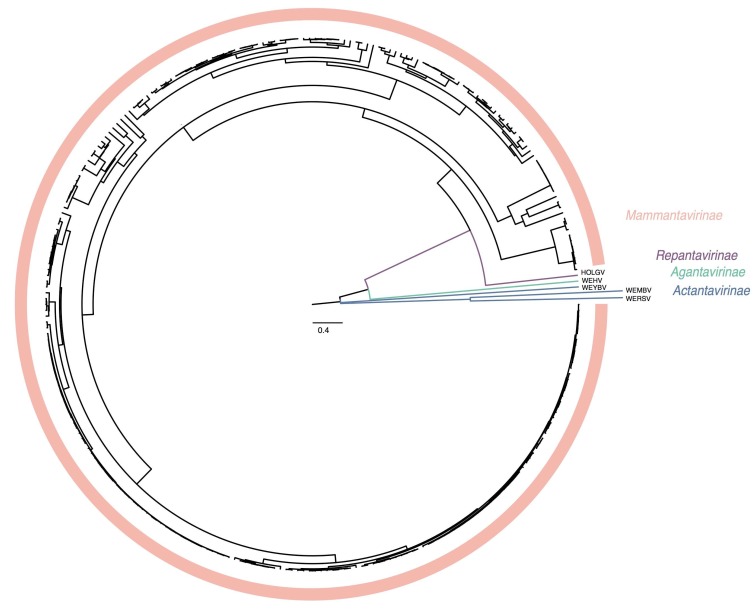
Bayesian inference of concatenated S and M segment-encoded protein amino acid sequences of *Hantaviridae*. A maximum clade credibility tree of the complete amino acid sequence of the hantavirid S and M segment-encoded proteins divides the family into four subfamilies.

The recent discoveries of hantaviruses in a wide spectrum of host species have significantly increased the known hantavirus diversity. Phylogenetic inference divides *Hantaviridae* in well-supported subclades (Figure 3). These taxonomic sub-groups are now better defined by the introduction of genera and subfamilies (Table 3).

### 3.5. Etymology of Taxa included in Hantaviridae

Subfamilies:

- *Actantavirinae:* derived from genus name *Actinovirus*, family name *Hantaviridae*, and subfamily suffix -*virinae*;
- *Agantavirinae:* derived from genus name *Agnathovirus*, family name *Hantaviridae*, and subfamily suffix -*virinae*;
- *Mammantavirinae:* derived from host class name Mammalia, family name *Hantaviridae*, and subfamily suffix -*virinae*;
- *Repantavirinae:* derived from genus name *Reptillovirus*, family name *Hantaviridae*, and subfamily suffix -*virinae*.

Genera

- *Actinovirus:* derived from host class Actinopterygii and genus suffix *-virus*;
- *Agnathovirus:* derived from host superclass Agnatha and genus suffix *-virus*;
- *Loanvirus:* derived from Lóngquán virus and genus suffix *-virus*;
- *Mobatvirus:* derived from mole and bat hosts and genus suffix *-virus*;
- *Orthohantavirus:* derived from Greek ὀρθός [orthós], meaning “straight,” historical genus *Hantavirus*, and genus suffix *-virus*;
- *Thottimvirus:* derived from Thottapalayam virus, Imjin virus, and genus suffix *-virus*;
- *Reptillovirus:* derived from host class Reptilia and genus suffix *-virus*.

### 3.6. Megataxonomy of Hantaviridae

A recent global phylogenetic analysis confirmed the monophyly of negative-sense RNA viruses [11]. A top taxonomic rank was introduced by the ICTV for all RNA viruses [80] including a phylum, 2 subphyla, and several classes for negative-sense RNA viruses [81,82]. The current megataxonomy of *Hantaviridae* is outlined in Table 4.

### 3.7. Etymology of Megataxa Relating to Hantaviridae

- *Riboviria*: derived from ribonucleic acid and realm suffix -*viria*;
- *Negarnaviricota*: derived from the Latin *Nega*, meaning “negative” *RNA*, and phylum suffix -*viricota*;
- *Polyploviricotina*: derived from Ancient Greek πολύπλοκος [polýplokos] for “complex” and subphylum suffix -*viricotina*;
- *Ellioviricetes*: derived from Richard Elliott, the late pioneer of bunyaviral molecular virology, and class suffix -*viricetes*;
- *Bunyavirales*: derived from Bunyamwera virus and order suffix -*virales*.

## 4. Future Taxonomic Perspectives

In 2020, hantavirid taxonomy will likely only change minimally, because only a single TaxoProp has been submitted by the 2019 submission deadline. This TaxoProp outlines the addition of one loanvirus species for the recently discovered Brno virus (BRNV) [83]. The megataxonomic placement of *Hantaviridae* will likely remain steady, but phylum *Negarnaviricota* will likely be included in the newly proposed kingdom “*Orthornavira*”.

Novel TaxoProps are already expected to be submitted by the next submission deadline in 2020 for the 2021 taxonomy cycle to accommodate several recently described putative mobatviruses [84,85]. Furthermore, the ICTV *Hantaviridae* Study Group is currently discussing whether hantavirids, for which coding-complete S+M+L genomic segment sequences are not available, ought to be declassified and whether hantavirid name abbreviations ought to be unique (and be changed if they are not). The ICTV *Hantaviridae* Study Group is also discussing how species “complexes” (species that harbor more than one member virus) could be resolved, and how hantavirid species names could be changed to Linnaean binomial names [86] should this become an ICTV requirement.

## 5. Discussion

The current hantavirid taxonomy (Table 5) is based upon concatenated amino acid sequences of S and M genomic segment-encoded proteins. To provide a more robust framework, ideally only coding-complete sequences of hantavirids should be used for any classification efforts, with various methods analyzing all segments. Unfortunately, very few hantavirus genomes have been sequenced fully, precluding such a robust taxonomic classification for now. Increased sequencing efforts of partially characterized hantavirids, some of them discovered decades ago, could substantially improve future taxonomic efforts. In many cases, obtaining missing sequence information is not challenging scientifically, as most historic hantavirids have been isolated in culture. However, owing to the high sequence diversity and saturation of informative sites, classification with inclusion of the M segment might become increasingly difficult as hantavirid diversity may be enormous. Such diversity is indicated by detection of more divergent hantavirids in metagenomic samples and in fish and reptiles. Although hantavirid interspecies segment reassortment is thought to be fairly limited, reassortment events have shaped hantavirid evolution [43,44,87] and may further complicate classification efforts. We are calling on the hantavirid research community to weigh in on these issues and to contribute to taxonomic efforts, including TaxoProp writing and submission, to achieve a taxonomy that best reflects hantavirid evolutionary relationships.

## Figures and Tables

**Table 1:** New hantavirid species according to the 2017 ICTV taxonomy [52].

New Hantavirid Species	Hantavirid	Hantavirid Abbreviation	Reference (s)	Isolate Used for Analysis
*Amga orthohantavirus* ^1^	Amga virus	MGAV	[38,53]	AH301
*Asama orthohantavirus*	Asama virus	ASAV	[33]	N10
*Asikkala orthohantavirus*	Asikkala virus	ASIV	[54]	CZ/Beskydy/412/2010/Sm
*Bowe orthohantavirus*	Bowé virus	BOWV	[55]	VN1512
*Bruges orthohantavirus*	Bruges virus	BRGV	[44]	BE/Vieux-Genappe/TE/2013/1
*Cao Bang orthohantavirus*	Cao Bằng virus	CBNV	[56]	3
*Choclo orthohantavirus*	Choclo virus	CHOV	[57]	MSB96073
*Dabieshan orthohantavirus*	Dàbiéshān virus	DBSV	[58]	Yǒngjiā-Nc-58
*Fugong orthohantavirus*	Fúgòng virus	FUGV	[59]	FG10
*Fusong orthohantavirus*	Fǔsōng virus	FUSV	[60]	Fǔsōng-Mf-682
*Imjin thottimvirus* ^2^	Imjin virus	MJNV	[61]	Cíxī-Cl-23
*Jeju orthohantavirus*	Jeju virus	JJUV	[62]	10-11
*Kenkeme orthohantavirus*	Kenkeme virus	KKMV	[63]	Fǔyuǎn-Sr-326
*Laibin mobatvirus* ^2^	Láibīn virus	LAIV	[64]	BT20
*Longquan loanvirus* ^2^	Lóngquán virus	LQUV	[43]	Lóngquán-Rs-32
*Luxi orthohantavirus*	Lúxī virus	LUXV	[65]	LX309
*Maporal orthohantavirus*	Maporal virus	MAPV	[66]	HV-97021050
*Montano orthohantavirus*	Montaño virus	MTNV	[67]	104/2006
*Necocli orthohantavirus*	Necoclí virus	NECV	[68]	HV-O0020002
*Nova mobatvirus* ^2^	Nova virus	NVAV	[69]	3483 (Te34)
*Oxbow orthohantavirus*	Oxbow virus	OXBV	[70]	Ng1453
*Quezon mobatvirus* ^2^	Quezon virus	QZNV	[33]	MT1720/1657
*Rockport orthohantavirus*	Rockport virus	RKPV	[71]	MSB57412
*Yakeshi orthohantavirus*	Yákèshí virus	YKSV	[60]	Yákèshí-Si-210

^1^*Amga orthohantavirus* was abolished in the most recent (2019) taxonomic release (see 1.3.3), ^2^ Genera *Thottimvirus*, *Mobatvirus* and *Loanvirus* were introduced in 2018 (see 1.3.4).

**Table 2:** New hantavirid species according to the 2019 ICTV taxonomy [32].

New Hantavirid Species	Hantavirid	Hantavirid Abbreviation	Reference	Isolate Used for Analysis
*Hagfish agnathovirus*	Wēnlǐng hagfish virus	WEHV	[7]	DHMMS23081
*Batfish actinovirus*	Wēnlǐng minipizza batfish virus	WEMBV	[7]	XQTMS16810
*Spikefish actinovirus*	Wēnlǐng red spikefish virus	WERSV	[7]	XTXMS70955
*Goosefish actinovirus*	Wēnlǐng yellow goosefish virus	WEYGV	[7]	XQTMS34106
*Seewis orthohantavirus*	Seewis virus	SWSV	[34]	EWS25
*Tigray orthohantavirus*	Tigray virus	TIGV	[72]	ET2121
*Gecko reptillovirus*	Hǎinán oriental leaf-toed gecko virus	HOLGV	[7]	LPXYC213122

**Table 3:** Classification overview of *Hantaviridae* in 2019 [32].

Subfamily	Genus	Number of Genus-Included Species	Number of Genus-Assigned Viruses	Host (s)
*Actantavirinae*	*Actinovirus*	3	3	Ray-finned fish
*Agantavirinae*	*Agnathovirus*	1	1	Jawless fish
*Mammantavirinae*	*Loanvirus*	1	1	Bats
	*Mobatvirus*	3	3	Bats or moles
	*Orthohantavirus*	36	58	Rodents or shrews
	*Thottimvirus*	2	2	Shrews
*Repantavirinae*	*Reptillovirus*	1	1	Reptiles

**Table 4:** Megataxonomic placement of *Hantaviridae*.

Taxonomic Rank	Taxon
Realm Kingdom Phylum	*Riboviria* Unassigned *Negarnaviricota*
Subphylum	*Polyploviricotina*
Class	*Ellioviricetes*
Order	*Bunyavirales*

**Table 5:** Classification of hantavirids in 2019 [32].

Subfamily	Genus	Species	Virus (Abbreviation)
*Actantavirinae*	*Actinovirus*	*Batfish actinovirus* *	Wēnlǐng minipizza batfish virus (WEMBV)
		*Goosefish actinovirus*	Wēnlǐng yellow goosefish virus (WEYGV)
		*Spikefish actinovirus*	Wēnlǐng red spikefish virus (WERSV)
*Agantavirinae*	*Agnathovirus*	*Hagfish agnathovirus* *	Wēnlǐng hagfish virus (WEHV)
*Mammantavirinae*	*Loanvirus*	*Longquan loanvirus* *	Lóngquán virus (LQUV)
	*Mobatvirus*	*Laibin mobatvirus*	Láibīn virus (LAIV)
		*Nova mobatvirus* *	Nova virus (NVAV)
		*Quezon mobatvirus*	Quezon virus (QZNV)
	*Orthohantavirus*	*Andes orthohantavirus*	Andes virus (ANDV)
			Castelo dos Sonhos virus (CASV)
			Lechiguanas virus (LECV = LECHV)
			Orán virus (ORNV)
		*Asama orthohantavirus*	Asama virus (ASAV)
		*Asikkala orthohantavirus*	Asikkala virus (ASIV)
		*Bayou orthohantavirus*	bayou virus (BAYV)
			Catacamas virus (CATV)
		*Black Creek Canal orthohantavirus*	Black Creek Canal virus (BCCV)
		*Bowe orthohantavirus*	Bowé virus (BOWV)
		*Bruges orthohantavirus*	Bruges virus (BRGV)
		*Cano Delgadito orthohantavirus*	Caño Delgadito virus (CADV)
		*Cao Bang orthohantavirus*	Cao Bằng virus (CBNV)
			Liánghé virus (LHEV)
		*Choclo orthohantavirus*	Choclo virus (CHOV)
		*Dabieshan orthohantavirus*	Dàbiéshān virus (DBSV)
		*Dobrava-Belgrade orthohantavirus*	Dobrava virus (DOBV)
			Kurkino virus (KURV)
			Saaremaa virus (SAAV)
			Sochi virus (SOCV)
		*El Moro Canyon orthohantavirus*	Carrizal virus (CARV)
			El Moro Canyon virus (ELMCV)
			Huitzilac virus (HUIV)
		*Fugong orthohantavirus*	Fúgòng virus (FUGV)
		*Fusong orthohantavirus*	Fǔsōng virus (FUSV)
		*Hantaan orthohantavirus* *	Amur virus (AMRV)
			Hantaan virus (HTNV)
			Soochong virus (SOOV)
		*Jeju orthohantavirus*	Jeju virus (JJUV)
		*Kenkeme orthohantavirus*	Kenkeme virus (KKMV)
		*Khabarovsk orthohantavirus*	Khabarovsk virus (KHAV)
			Topografov virus (TOPV)
		*Laguna Negra orthohantavirus*	Laguna Negra virus (LANV)
			Maripa virus (MARV)
			Rio Mamoré virus (RIOMV)
		*Luxi orthohantavirus*	Lúxī virus (LUXV)
		*Maporal orthohantavirus*	Maporal virus (MAPV)
		*Montano orthohantavirus*	Montaño virus (MTNV)
		*Necocli orthohantavirus*	Necoclí virus (NECV)
		*Oxbow orthohantavirus*	Oxbow virus (OXBV)
		*Prospect Hill orthohantavirus*	Prospect Hill virus (PHV)
		*Puumala orthohantavirus*	Hokkaido virus (HOKV)
			Muju virus (MUJV)
			Puumala virus (PUUV)
		*Rockport orthohantavirus*	Rockport virus (RKPV)
		*Sangassou orthohantavirus*	Sangassou virus (SANGV)
		*Seewis orthohantavirus*	Seewis virus (SWSV)
		*Seoul orthohantavirus*	gōu virus (GOUV)
			Seoul virus (SEOV)
		*Sin Nombre orthohantavirus*	New York virus (NYV)
			Sin Nombre virus (SNV)
		*Thailand orthohantavirus*	Anjozorobe virus (ANJZV)
			Serang virus (SERV)
			Thailand virus (THAIV)
		*Tigray orthohantavirus*	Tigray virus (TIGV)
		*Tula orthohantavirus*	Adler virus (ADLV)
			Tula virus (TULV)
		*Yakeshi orthohantavirus*	Yákèshí virus (YKSV)
	*Thottimvirus*	*Imjin thottimvirus*	Imjin virus (MJNV)
		*Thottapalayam thottimvirus* *	Thottapalayam virus (TPMV)
*Repantavirinae*	*Reptillovirus*	*Gecko reptillovirus* *	Hǎinán oriental leaf-toed gecko virus (HOLGV)
